# Effectiveness of *Viola* Flower Syrup Compared with Polyethylene Glycol in Children with Functional Constipation: A Randomized, Active-Controlled Clinical Trial

**DOI:** 10.1155/2021/9915289

**Published:** 2021-09-09

**Authors:** Sara Tavassoli, Kambiz Eftekhari, Mehrdad Karimi, Ali Ghobadi, Mohsen Shati, Amir Naddaf, Alireza Abbassian

**Affiliations:** ^1^Department of Traditional Medicine, School of Persian Medicine, Tehran University of Medical Sciences, Tehran, Iran; ^2^Pediatric Gastroenterology and Hepatology Research Center, Pediatrics Department, Bahrami Children's Hospital, Tehran University of Medical Sciences, Tehran, Iran; ^3^Department of Traditional Pharmacy, School of Persian Medicine, Iran University of Medical Sciences, Tehran, Iran; ^4^Mental Health Research Center (MHRC), School of Behavioral Sciences and Mental Health, Tehran Institute of Psychiatry, Iran University of Medical Sciences, Tehran, Iran; ^5^School of Public Health, Department of Epidemiology, Iran University of Medical Sciences, Tehran, Iran; ^6^Department of Pediatrics, School of Medicine, Maternal, Fetal, and Neonatal Research Center, Family Health Research Institute, Vali Asr Hospital, Imam Khomeini Hospital Complex, Tehran University of Medical Sciences, Tehran, Iran

## Abstract

**Background:**

Functional constipation (FC) is a health concern that is prevalent in the pediatric population. It lowers the quality of life and increases the probability of comorbidities. As a complementary modality, herbal medicine has been considered useful in a variety of conditions. Persian medicine (PM) resources mention the *Viola* flower as an effective herb in treating constipation. The purpose of the current trial was to evaluate the efficacy of *Viola* flower syrup (VFS) compared with polyethylene glycol (PEG) in children with functional constipation.

**Methods:**

This randomized, active-controlled, single-center trial was conducted on 140 children aged between 4 and 10 years with confirmed FC according to Rome III criteria. Participants were randomly assigned to receive either VFS or PEG for four weeks. Independent *t*-test and general linear model (GLM) repeated measures analysis of variance were used to determine the intergroup difference, and paired sample *t*-test was used to evaluate the intragroup difference.

**Results:**

After four weeks of intervention, 133 individuals (66 in VFS and 67 in the PEG group) were analyzed. Results of both groups demonstrated significant improvement in all measured criteria at the end of the study compared to baseline (*P* < 0.001). No significant difference was observed between the two groups at baseline or at the end of the study (*P* > 0.05), except for fecal retention at baseline (*P*=0.028). Participants in the PEG group experienced more side effects compared to the VFS group.

**Conclusion:**

The findings of this investigation indicated that VFS is an effective and relatively safe medication to be used in the treatment of pediatric FC.

## 1. Introduction

Constipation is a great health challenge in childhood. The precise prevalence is hard to determine, but it is estimated that about 0.7% to 29.6% of children suffer from constipation [1]. The majority of affected children (90%) have functional constipation (FC) [2]. The etiology of FC is multifactorial and results from the interaction of several factors such as genetics, lifestyle (for instance, diet, physical activity, and withholding behavior), and psychological factors (such as anxiety, stress, autism, and ADHD) [3, 4]. Symptoms of FC in children consist of hard, infrequent bowel movements, bloating, abdominal pain, and fecal incontinence [3]. Constipation not only has negative effects on children's quality of life [5] but also increases the risk of comorbidities, including depression, anxiety, influenza, otitis media, and asthma, and puts a heavy financial burden on the society [6, 7].

Various approaches, such as education, alteration in dietary habits, behavioral intervention, and pharmacotherapy, have been used to treat constipation [8]. Different types of laxatives, such as stimulant, bulk, and emollient agents, are prescribed for this condition [9, 10]. Polyethylene glycol (PEG) is a laxative usually considered as the first-line treatment based on ESPGHAN/NASPGHAN guidelines [11]. This polymer does not metabolize in the intestines, causes an osmotic gradient, and subsequently keeps fluids in the lumen of the colon, thereby softening and loosening the stools to accelerate defecation [12]. Laxatives are accompanied by adverse side effects such as abdominal pain and bloating, and their efficacy may dwindle over time [13]. Furthermore, some prospective trials have shown that 50% of children still have constipation complaints after five years of intensive medical and behavioral treatment [14]. Due to high expenditure, low efficacy, and undesirable side effects of conventional drugs, there has been a growing tendency towards complementary and alternative medicine (CAM) in recent years [14]. It is estimated that approximately 36.4% of children use CAM, of which 24.1% use it to treat constipation [15].

One of the most popular methods of CAM is herbal medicine. Persian medicine (PM) suggests various approaches for treating diseases, amongst which herbs are the most prevalent component [16]. *Viola*, scientifically known as *Viola odorata,* is a medicinal herb used by Persian physicians, such as Avicenna (980–1037 AD) and Haly Abbas (930–994 AD), to treat different conditions. A number of studies have reported antioxidant, anti-inflammatory, anticancer, sedative, diuretic, and laxative properties for this herb [17]. PM resources recommend *Viola odorata* as an effective treatment for pediatric constipation, but the efficacy and safety of this medicinal herb have not been proved in modern studies.

Considering complications of conventional medicine and insufficient efficacy, this clinical trial was designed to assess the effectiveness and safety of *Viola* flower syrup (VFS) as a natural laxative, compared to PEG, as a standard treatment, in 4–10-year-old children with FC.

## 2. Methods

### 2.1. Trial Design

This study was a 4-week, randomized, active-controlled, parallel trial performed in Bahrami Pediatric Hospital affiliated to Tehran University of Medical Sciences from May 2018 to May 2019.

The study design was approved by the Review Board and the Ethical Committee of Tehran University of Medical Sciences (TUMS) (no. IR.TUMS.VCR.REC.1396.4668) and performed in accordance with the Declaration of Helsinki and its subsequent revisions. The trial was registered at the Iranian Registry of Clinical Trials on April 13, 2018 (https://www.irct.ir/trial/30343; registration no. IRCT20180305038968N1). Written consent was obtained from children's parents before recruitment. The purpose, procedure, advantages, and disadvantages of the study were presented to parents, and they were aware that they were able to withdraw from the intervention at any time.

### 2.2. Participants

Participants were selected from children aged between 4 and 10 years with FC confirmed by a gastrointestinal pediatrician based on Rome III criteria [18]. FC was described as suffering from constipation for equal or over two months plus at least two of the following criteria: ≤ 2 bowel movements per week, ≥ 1 episode of fecal incontinence per week, history of retentive posturing or excessive volitional stool retention, history of pain or hard bowel movements, presence of a large fecal mass in the rectum, and history of large-diameter stools that may obstruct the toilet. Exclusion criteria consisted of organic constipation due to disorders such as hypothyroidism, Hirschsprung's disease, chronic intestinal pseudo-obstruction, presence of other chronic diseases such as asthma, using medications that cause constipation, having used drugs to treat constipation during the last month, history of gastrointestinal surgery, neurological abnormality, anatomical abnormality, and history of allergy to herbs or intolerance to PEG. Participants exited the study if they reacted to intervention, did not follow instructions correctly, and were not willing to continue intervention or if constipation worsened during the study.

### 2.3. Sample Size Estimation

G^*∗*^Power software (version 3.1.9) [19, 20] was used to estimate the necessary sample size before the study. To achieve a moderate effect size (*d* = 0.5) with a static power of 0.8 at a significant level of 0.05, a total of 64 children were required in each group. However, this was increased to 70 subjects to account for a 10% dropout rate.

### 2.4. Randomization

Eligible children were randomly allocated to one of the therapeutic groups with a 1 : 1 ratio by the permuted randomization method (with block sizes of four) to receive either VFS or PEG, with no subsequent crossover. A random number list generated by using a computer was used to assign participants to two arms. The researcher conducting randomization was not involved in other parts of the study.

### 2.5. Intervention

Intervention included oral administration of PEG solution 40% (1 g/kg/day) or VFS (5 cc 3 times per day) [21]. Participants in each group received one of these drugs for four weeks. To prepare a 40% PEG solution, 400 g of the PEG powder was dissolved in 600 mL water which was added gradually to adjust the volume to 1000 mL. This solution was sterilized at an autoclave temperature and then kept at refrigerator temperature. To prepare VFS, 1 kg of dried *Viola* flower was soaked in 6 liters of water at room temperature for 4 hours. Subsequently, while adding some sugar for consistency, it was heated to boil gently for an hour and then filtered. The filtrate solution was 4 liters. The PEG solution and VFS were delivered to the participants in similar containers so that they were not detectable and there was no contact between patients. Both drugs were manufactured by the Department of Traditional Pharmacology, School of Persian Medicine, TUMS.

### 2.6. Outcomes

The outcomes of the study were gathered by a nurse who was not aware of group allocations. A sociodemographic questionnaire was used to obtain the general characteristics of participants. This questionnaire comprised data such as age, sex, weight, height, and duration of constipation.

The primary outcome was the difference between the response to treatment in the VFS and PEG groups following four weeks of intervention. Response to treatment was defined as improvement in constipation symptoms based on Rome III criteria [18, 22]. The children's parents completed this questionnaire. FC was assessed at five time points, including T0 (baseline), T1 (end of the 1^st^ week), T2 (end of the 2^nd^ week), T3 (end of the 3^rd^ week), and T4 (end of the 4^th^ week). The first assessment included an in-person interview, and other examinations were performed over the phone. In each remote visit, children were also checked for exit criteria, correct consumption of medications, and potential side effects. The primary and secondary outcomes included stool consistency, defecation frequency, hard stools, painful defecation, fecal retention, and fecal soiling. The efficacy of treatment at the end of the study was compared with the start of the study and between groups. Treatment was considered effective if there was a significant difference in comparison with the beginning of the study. Parents could call the researcher for any questions at any time and were educated to report potential problems immediately.

### 2.7. Statistical Analyses

SPSS software version 22 (SPSS Inc., Chicago, IL, USA) for Windows was used to analyze data. Initially, baseline characteristics were compared between groups. For quantitative characteristics, an independent *t*-test was applied, and data were presented as mean ± SD. The *χ*^2^ test was used for qualitative factors and presented as percentages. Rome III criteria, including stool consistency, defecation frequency, hard stool, painful defecation, fecal retention, and fecal soiling, were compared between groups by an independent *t*-test at the initiation and end of the study. General linear model (GLM) repeated measures analysis of variance was performed to compare VFS and PEG across the five time points of baseline, intervention period (week 1, week 2, and week 3), and the end of the study (week 4) in the intervention vs. control groups. In all the models, duration of constipation and weight were included as covariates. A paired sample *t*-test was also used to compare the mean of variables before and after treatment in each group. To compare side effects between groups, chi-square test was performed.

## 3. Results

**Figure 1:**
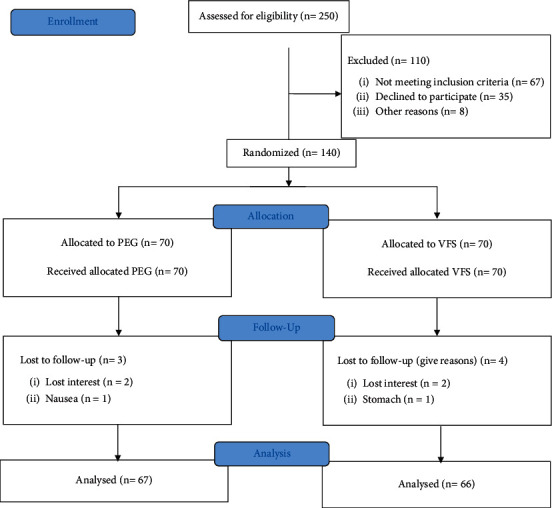
Flow diagram of the study.

Among 250 children referred to the hospital, 140 patients who met the inclusion criteria were chosen to enter to study. Participants were subsequently assigned randomly to one of the VFS and PEG groups. Three participants in the PEG (2 unwilling and 1 due to vomiting) and 4 in VFS (3 unwilling and 1 due to stomach ache) withdrew from the study. Finally, 133 individuals completed the intervention and were followed for 4 weeks (Figure 1).  Table 1 indicates the general characteristics of children in two treatment groups. There was no significant difference in terms of age, height, and sex, but participants in the VFS group weighed more and suffered from constipation for a longer time compared with the PEG group.

**Figure 2:**
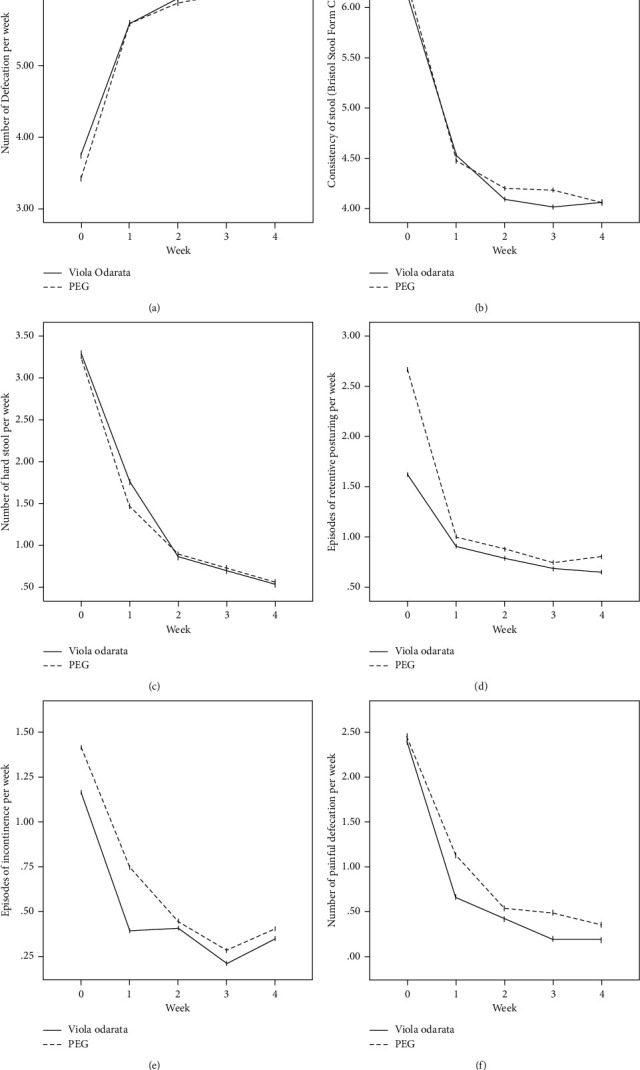
Defecation frequency (a), fecal incontinence (b), hard stool (c), episodes of retentive posturing (d), episodes of inconsistence (e), and painful defecation (f) in the two treatment groups of *Viola* flower syrup and PEG in children with functional constipation before and after intervention.

In terms of the Rome III criteria, there was no significant difference between groups at the baseline of the study in any constipation symptoms except fecal retention (*P*=0.028). After the initiation of intervention, all symptoms improved in both VFS and PEG groups gradually, as shown in Figure 2. Both medications had similar efficacies, and constipation symptoms were significantly relieved in all patients at the end of the study. However, no significant difference was observed between the two groups (Table 2).

The incidence of side effects was significantly different between the two groups, with the PEG group reporting more complaints, such as abdominal pain, loose stool, nausea, vomiting, and unpleasant taste (Table 3).

## 4. Discussion

This single-center trial was designed to assess the efficacy of VFS compared with PEG in treating pediatric FC. *Viola odorata* is recommended by Persian scholars to manage constipation. However, to our knowledge, no randomized trial has been conducted to evaluate the effect of this medicinal herb on FC in children.

The findings of this trial confirmed that PEG improves constipation symptoms in children. A comparative study on infants and children concluded that PEG was more effective in treating constipation, with a lower risk of side effects compared with lactulose [23]. PEG is an unabsorbable and unmetabolizable polymer (less than 1%) that enhances fluid retention in the intestine. However, this medication is usually accompanied by unwanted complaints, including nausea, abdominal pain, electrolyte imbalance, flatulence, and fecal incontinence [24].

*Viola odorata* is a medicinal plant that belongs to the family Violaceae and is native to Asia, North Africa, and Europe [25]. Based on traditional references, it is claimed that *Viola odorata* has therapeutic features in treating cough, fever, common cold, headache, insomnia, epilepsy, constipation, palpitation, dyspnea, dysuria, and skin diseases [26]. The effect of *Viola odorata* products on different conditions has been assessed in numerous studies. Several of these studies have been conducted on a pediatric population. An experimental study on rats demonstrated antihypertensive and antidyslipidemic effects for the *Viola odorata* leaf extract [27]. Moreover, Feyzabadi and colleagues conducted a trial on patients with chronic insomnia and concluded that *Viola* oil could induce sleep without notable side effects [28]. A double-blind, randomized controlled trial by Qasemzadeh et al. was conducted on 182 children aged 2–12 years with intermittent asthma. This study demonstrated that VFS plus usual treatment is significantly effective in suppressing asthma coughs compared with placebo [21]. Another clinical trial revealed that *Viola* oil is a safe and effective therapy in controlling fever in febrile neutropenic children [29].

According to the current study results, VFS is as effective as PEG in relieving constipation symptoms, with significantly fewer side effects. Some animal studies have investigated the laxative and prokinetic impact of different types of violet, the results of which were in accordance with our findings. Vishal et al. conducted a study on rats to evaluate the laxative effects of different forms of *Viola odorata*. They found that 200 mg/kg alcoholic extract and 400 mg/kg aqueous extract of violet have remarkable laxative effects. On the contrary, butanolic and aqueous extracts at both doses of 200 mg/kg and 400 mg/kg led to significant gastrointestinal motility [30]. In another study on mice, results revealed that the crude methanolic extract of *Viola betonicifolia* could improve bowel movement through laxative (at the dose of 30 and 100 mg/kg) and prokinetic (at the amount of 50 and 100 mg/kg) effects in mice [31]. However, the exact anticonstipation mechanism of VFS has not been elucidated. This medicinal herb may improve constipation symptoms via laxative and prokinetic activities.

Besides strength points such as appropriate sample size, valid and reliable tools, and assessment of outcomes and side effects at the end of each week, this study has several limitations. The clinician was not blinded to the treatment, and the dosage of medications was not similar. The intervention period was short, and patients were not followed up after termination of intervention. Moreover, sugar, as an ingredient of syrup, might have positive effects on constipation, leading to bias. Also, various factors such as diet, genetics, physical activity, and other lifestyle factors could influence constipation but are not considered as confounders.

Our findings indicated that similar to conventional treatment, VFS is an effective and relatively safe medication in children with FC. However, more studies should be conducted on the mechanism and safety of this herb, especially in allergic patients.

## Figures and Tables

**Table 1:** Baseline characteristics of participants.

Characteristics	VFS (*n* = 70)	PEG (*n* = 70)
Age (year)	7.01 ± 2.23	6.29 ± 2.13
Sex, male, *n* (%)	35 (50)	34 (48)
Weight (kg)	24.15 ± 7.84	21.11 ± 7.10
Height	119.88 ± 19.28	115.90 ± 13.79
Duration of symptoms (months)	42.34 ± 32.84	28.71 ± 26.18

**Table 2:** Results of the KOOS questionnaire between intervention (VFS) and control (PEG) groups.

Variables	VFS (*n* = 66)	PEG (*n* = 67)	*P* value^a^
	Mean ± SD	Mean ± SD	
*Stool consistency*			
Before treatment	6.12 ± 0.80	6.23 ± 0.72	0.331
After treatment	4.06 ± 0.39	4.06 ± 0.78	0.504
*P* value^b^	<0.001	<0.001	

*Defecation frequency*			
Before treatment	3.74 ± 1.65	3.42 ± 1.80	0.294
After treatment	6.01 ± 1.47	6.16 ± 1.33	0.536
*P* value	<0.001	<0.001	

*Hard stool*			
Before treatment	3.30 ± 1.61	3.25 ± 1.72	0.434
After treatment	0.53 ± 1.13	0.56 ± 1.15	0.993
*P* value	0.001	0.001	

*Painful defecation*			
Before treatment	2.24 ± 1.56	2.28 ± 1.68	0.873
After treatment	0.25 ± 1.01	0.40 ± 0.94	0.376
*P* value	<0.001	<0.001	

*Fecal retention*			
Before treatment	1.62 ± 2.41	2.67 ± 3.00	0.028
After treatment	0.65 ± 1.85	0.80 ± 1.75	0.625
*P* value	0.001	0.001	

*Fecal soiling*			
Before treatment	1.16 ± 2.13	1.41 ± 2.35	0.521
After treatment	0.34 ± 1.27	0.40 ± 1.25	0.804
*P* value	0.001	0.001	

^a^*P* value is calculated by the independent *t*-test. ^b^*P* value is calculated by the paired *t*-test.

**Table 3:** Comparison of side effects of the treatment in the groups.

Groups	Abdominal pain	Loose stools	Nausea	Vomiting	Unpleasant taste	*P* value
VFS	1 (1.4%)	0	0	0	1 (1.4%)	0.03
PEG	6 (8.6%)	2 (2.9%)	2 (2.9%)	1 (1.4%)	2 (2.9%)	

## Data Availability

The data used to support the findings of this study are available from the authors.
